# Mediastinal Actinomycosis After Esophagogastroduodenoscopy

**DOI:** 10.7759/cureus.8423

**Published:** 2020-06-03

**Authors:** Connor S Thrun, Joshua Boster, Nathan Jansen, David Dado, Jason Okulicz

**Affiliations:** 1 Internal Medicine, Brooke Army Medical Center, Fort Sam Houston, San Antonio, USA; 2 Infectious Disease, Brooke Army Medical Center, Fort Sam Houston, San Antonio, USA; 3 Nephrology, Brooke Army Medical Center, Fort Sam Houston, San Antonio, USA

**Keywords:** egd, actinomycosis

## Abstract

Actinomycosis is an uncommon bacterial infection that presents as an indolent, progressive disease that can affect multiple organ systems. We describe the case of a 66-year-old female with end-stage renal disease who presented to the emergency department after developing acute dyspnea and chest pain two weeks after undergoing a diagnostic esophagogastroduodenoscopy (EGD). A CT scan was obtained that revealed a large mediastinal mass, which was initially concerning for a potential malignancy. Biopsy of the mass and Gram stain was consistent with mediastinal actinomycosis. The patient was subsequently treated with an extended course of antibiotics that resulted in significant clinical improvement. Previously reported cases describing a correlation between EGD and mediastinal actinomycosis have been associated with invasive procedures such as esophageal stent placement and transesophageal biopsy. We describe a case of an uncommon infectious complication of a diagnostic EGD that was not associated with intentional mucosal disruption.

## Introduction

*Actinomyces *species are branching, filamentous Gram-positive bacilli that live on mucosal surfaces as part of the normal flora. This species typically colonizes the oral cavity but has been found throughout the gastrointestinal tract. Infection can occur when the mucosal barrier is disrupted by trauma allowing the bacteria to invade into the deeper tissue [1]. Depending on the site of disruption, actinomycosis can affect multiple organ systems including the esophagus, thoracic cavity, abdomen, and pelvis. The mediastinum is a particularly rare location for this infection [2]. We describe a case of mediastinal actinomycosis, which resulted as a probable complication of an esophagogastroduodenoscopy (EGD).

## Case presentation

A 66-year-old female with a history of diabetes, hypertension, Roux-en-Y gastric bypass and end-stage renal disease (ESRD) presented to the emergency department with an acute onset of dyspnea. Two weeks prior to her presentation, the patient had undergone an uncomplicated diagnostic EGD with trans-duodenal endoscopic ultrasound-guided biopsy of a known pancreatic cyst.

The patient presented with one day of sudden shortness of breath and chest pain. Initial investigation of her acute dyspnea included a CT pulmonary angiogram that found multiple subsegmental pulmonary emboli. She was subsequently found to have a 7.7 x 3.4 x 6.7 cm sub-carinal soft tissue mass. The patient denied fever, shortness of breath, chest pain, weight loss, or other symptomatology prior to the day of presentation.

Laboratory data on admission was notable for a mild neutrophil predominant leukocytosis of 11.98 x 10^3^ cells/µL (normal: 3.4-9.8 x 10^3^ cells/µL) and an elevated ferritin level measuring 2812 ng/mL (normal: 30-400 ng/mL). Throughout her hospitalization, the patient had a persistent leukocytosis, with a peak of 17.01 x 10^3^ cells/µL. Blood cultures were obtained on admission and had no growth. The patient remained afebrile during her hospitalization.

**Figure 1:**
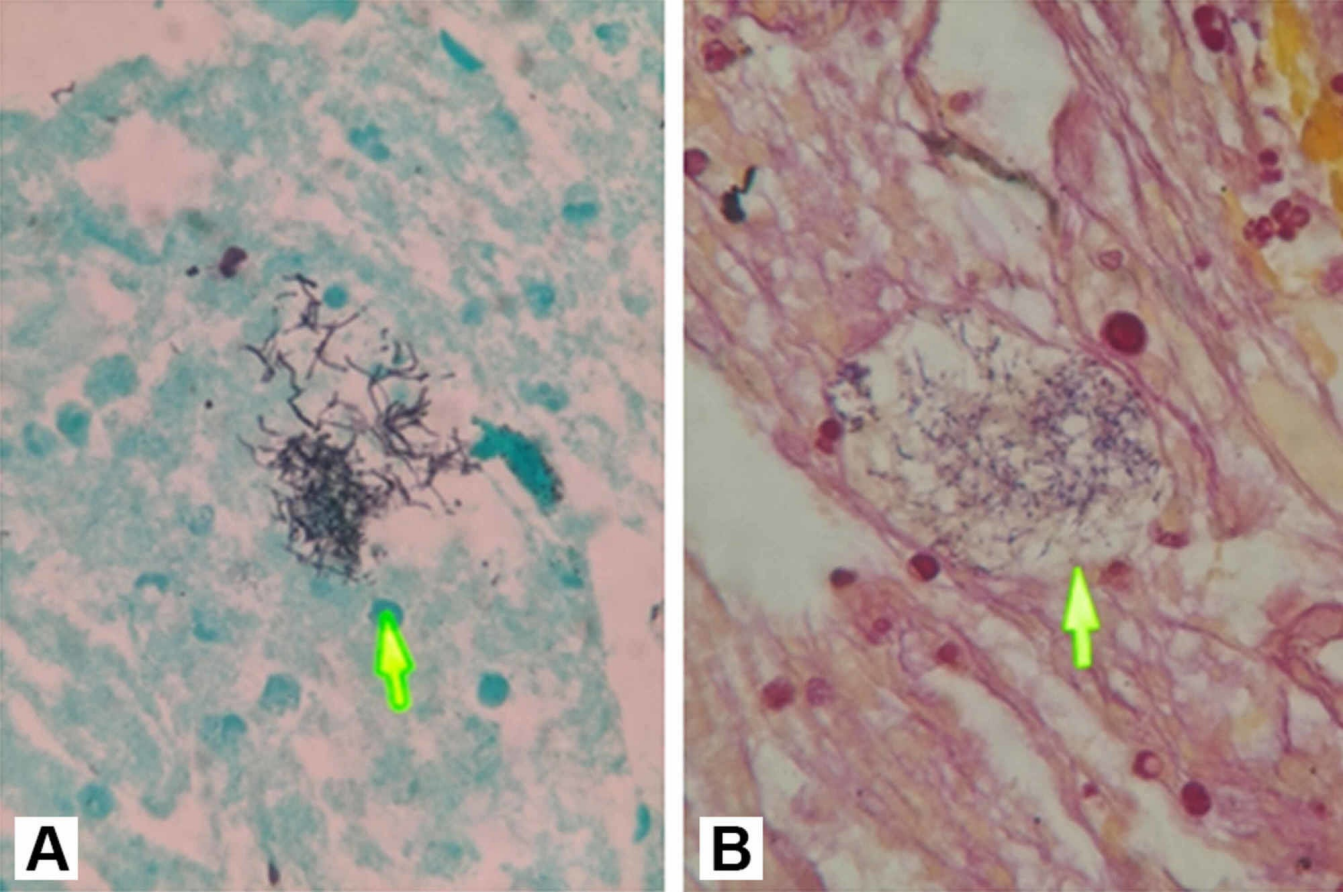
(A) Grocott-Gomori's stain and (B) Brown-Hopps (B) stain showing filamentous bacteria (arrows)

After starting anticoagulation to address her pulmonary emboli, focus turned to the diagnosis of the newly discovered sub-carinal mass. The mass was initially concerning for malignancy given her history of pancreatic cysts, family history of pancreatic cancer, and pulmonary emboli on presentation. A biopsy was obtained via trans-esophageal endoscopic ultrasound. Histopathology of the tissue revealed a non-acid fast, Gram-positive filamentous bacterium consistent with *Actinomyces *on Grocott-Gomori’s stain and confirmed on Brown-Hopps stain. There were abundant neutrophils and necrotic debris suggestive of an abscess. No sulfur granules were seen, and no tissue culture was collected. No malignant cells were identified (Figures 1A, 1B). The patient was diagnosed with mediastinal actinomycosis and infectious disease consultation was done.

Renally dosed intravenous vancomycin and amoxicillin/clavulanate potassium were initiated. Both antibiotics were administered for a five-week course, and then transitioned to amoxicillin monotherapy with a plan to continue until resolution of the mass. Given the location of the abscess and the patient’s comorbidities, she was determined to not be a candidate for percutaneous or surgical drainage.

**Figure 2:**
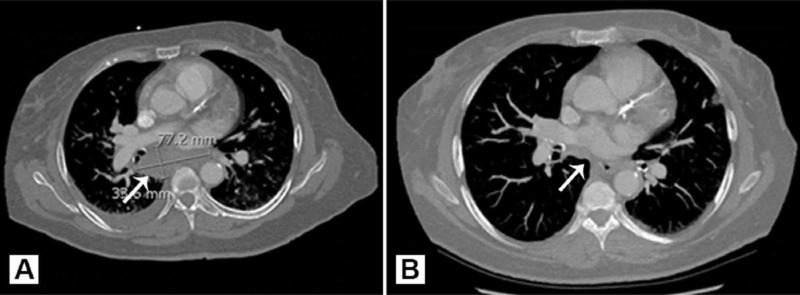
CT imaging of sub-carinal medialstinal mass (arrows) at (A) presentation and (B) following five months of therapy

After five months of treatment, the abscess reduced in size from 7.7 x 3.4 x 6.7 cm to 1.1 x 1.8 x 3.7 cm (Figures 2A, 2B). CT of the chest was obtained at three-month intervals to evaluate treatment response, and therapy is currently ongoing.

## Discussion

The presenting signs and symptoms of actinomycosis are nonspecific and presentations can vary depending on the site of infection. General symptoms include fever, weight loss, and cough [1]. Mediastinal actinomycosis has been reported to be a rare cause of superior vena cava syndrome, and has also been associated with exudative pleural effusions [3,4]. Furthermore, actinomycosis can often be confused for malignancy given the commonality of its presentations [1].

Our patient presented with chest pain and dyspnea; however, these symptoms are likely well-explained by the pulmonary emboli given her acute onset of symptoms. She was otherwise asymptomatic prior to her acute illness showcasing the indolent nature of this disease. The patient also had a small pleural effusion noted on imaging; however, this can be explained by her ESRD and chronic hypervolemia as it improved with dialysis.

*Actinomyces *exists commensally within the gastrointestinal tract but has the potential for infectious transformation upon translocation through the mucosal layer. This typically occurs via trauma or surgical procedures [1]. A prior report described a case of mediastinal actinomycosis that occurred three weeks following the placement of an esophageal stent for treatment of a fistula, in a patient with esophageal cancer, which illustrates the potential for *Actinomyces *to translocate within the mediastinum following procedural intervention within the esophagus [5]. Our patient underwent an EGD with trans-duodenal fine needle aspiration of a pancreatic cyst two weeks prior to her presentation, which is consistent with the timeline described in the study above; however, this procedure did not involve invasive intervention within the esophagus. We cannot definitively conclude that the abscess developed as a result of the procedure, but we hypothesize that bacterial translocation or an unrecognized microperforation introduced the bacteria into the mediastinum during the EGD. Our patient also had a history of a Roux-en-Y gastric bypass that may have resulted in increased risk for procedural complications given the associated anatomical changes.

Although we presume the esophagus was the source of our patient’s mediastinal infection, another potential etiology would include pulmonary actinomycosis with mediastinal spread. Pulmonary actinomycosis is commonly caused by aspiration, and risk factors include alcoholism, poor dental hygiene, prior pulmonary disease, and immunodeficiency [6]. Pulmonary actinomycosis typically presents on imaging as central areas of low attenuation within a consolidation and adjacent thickening of the pleura [2]. Our patient, however, had none of these risk factors and had no evidence of lung involvement on imaging, therefore, we believe that to be unlikely.

Mediastinal actinomycosis can be challenging to diagnose and requires histopathologic examination and culture to confirm the diagnosis, given the broad differential associated with mediastinal masses. Typical histopathologic findings include sulfur granules and Gram-positive branching filaments with surrounding inflammatory cells. *Actinomyces *is well known to be susceptible to most beta-lactam antibiotics. Intravenous penicillin followed by an extended period, typically months, of oral antibiotics is the first-line therapy for severe infections [1,6]. However, *Actinomyces *is generally susceptible to vancomycin and the ability to maintain therapeutic concentrations with intermittent dosing after dialysis makes vancomycin an ideal parenteral drug in dialysis patients. Our patient experienced a good clinical and radiographic response with vancomycin and amoxicillin/clavulanate potassium combination therapy followed by prolonged amoxicillin monotherapy. There was a significant decrease in the size of the abscess over the five-month course of treatment. Surgical debulking and percutaneous drainage of the abscess were considered initially, but deferred given the patient's comorbidities, ongoing need for systemic anticoagulation in the setting of pulmonary emboli, and the anatomic location.

## Conclusions

This case illustrates the potential for mediastinal actinomycosis to occur after an otherwise uncomplicated EGD. While there have been few cases of mediastinal actinomycosis described after invasive EGD procedures, this case involved no direct procedure or trauma of the esophagus during the EGD. Therefore, a microperforation in the esophagus during the EGD should be considered as a cause of translocation of *Actinomyces *from the esophagus into the mediastinum. As actinomycosis can be mistaken for a malignancy, biopsy and histopathologic examination are essential for the diagnosis. In this patient, prolonged antibiotic therapy without percutaneous or surgical intervention has resulted in significant clinical improvement.
